# *Peganum harmala* L. Intoxication in a Pregnant Woman

**DOI:** 10.1155/2014/783236

**Published:** 2014-05-14

**Authors:** Mohamed Adnane Berdai, Smael Labib, Mustapha Harandou

**Affiliations:** Child and Mother Intensive Care Unit, University Hospital Hassan II, Sidi Hrazem Avenue, 30000 Fes, Morocco

## Abstract

*Peganum harmala* L. is a plant widely distributed in the Mediterranean region. It is commonly used in traditional medicine in Morocco as sedative and abortifacient but exposes users to the risk of overdose and poisoning. The pharmacologically active compounds of this plant include a number of **β**-carboline and quinazoline alkaloids responsible of its pharmacological and toxicological effects. We report the case of a 24-year-old woman, 22 weeks pregnant, intoxicated with the seeds of *Peganum harmala* L. On admission, she had disturbance of consciousness, uterine contraction, and oliguria. Laboratory tests revealed renal failure and liver injury, and she benefited then from hemodialysis. During hospitalization, she was intubated after deterioration of consciousness and presented a spontaneous expulsion of the fetus. After extubation, she kept unusual sequelae: cerebellar ataxia and peripheral polyneuropathy. Physicians in regions using *Peganum harmala* L. as traditional medicine must be able to detect symptoms of its toxicity, in order to establish early gastrointestinal decontamination. The prognosis of this intoxication is variable; most cases can be managed successfully; but in high doses of intoxication, evolution can be fatal.

## 1. Introduction 

*Peganum harmala* L. (*P. harmala*), also known as Harmal or Syrian rue, is a perennial herbaceous, glabrous plant that grows in semiarid conditions, steppe areas, and sandy soils, native to eastern Mediterranean region. The plant is widely distributed and used as medicinal plant in Central Asia, North Africa, and Middle East [1]. The root and seeds contain several alkaloids that are pharmacologically active and responsible of their effect. The plant is used traditionally as an emmenagogue and an abortifacient agent in the Middle East and North Africa [2, 3]. We report the case of an intoxication of* P. harmala* in a pregnant woman, used as an abortive agent, and discuss the particularity of this rare reported intoxication.

## 2. Case Report

We report the case of a 24-year-old woman, without any medical history, 22 weeks pregnant, who is supposed to have taken a great quantity of seeds of* P. harmala* in order to provoke abortion as considered in traditional Moroccan remedy. On admission, she had disturbance of consciousness; the clinical examination revealed a Glasgow coma scale (GCS) at 12/15; the pupils were equal and reactive to light; she was afebrile, hemodynamically stable with heart rate of 60 pulse/min and arterial pressure of 110/70 mmHg. She presented also tachypnea with respiratory rate of 30/min; the SpO2 was 95% under oxygen facial mask at a flow rate of 6 L/min. Her urine was very concentrated; she had also uterine contraction.

The blood cell count and coagulation test were normal. Renal and liver function tests revealed a renal failure and a liver injury: blood urea nitrogen at 2.59 g/L, creatinine at 10.03 mmol/L, L-aspartate aminotransferase (AST) level at 83 IU/L (*N* < 40), L-alanine aminotransferase (ALT) at 245 IU/L (*N* < 45), and alkaline phosphatase (APL) at 544 IU/L (*N* < 165). The prothrombin time, total bilirubinemia level, and proteinuria were normal. The cardiac enzymes were elevated, the troponine was at 0.47 ng/mL, and creatine phosphokinase-MB was at 39 IU/L, associated with a normal electrocardiography. Radiologic investigations including chest radiography, cerebral magnetic resonance imaging, and kidney ultrasound were normal. Fetal ultrasound showed a vital fetus of 22 weeks of pregnancy.

Since the delay between intoxication and admission to the hospital exceeded 6 hours, the gastric lavage was not performed and activated charcoal was not administered. The treatment was only symptomatic including oxygen therapy, cimetidine, infusion of normal saline, and 5% dextrose solution. Unfortunately, we had not adequate laboratory facility to detect alkaloids in blood; the toxicological analyses searched only associated drug intoxications that alter consciousness (benzodiazepine, morphine) and were negative. Meanwhile, hemodialysis was performed secondary to the decrease of the urine output to 0.4 mL/kg/h, and after that, the patient shifted to critical care unit.

The day after, the patient showed a deterioration of GCS score who became at 10, associated to hypoventilation, stagnation of bronchial secretions, and hypercapnia in gazometry. She was then intubated, artificially ventilated, and kept sedated by a continuous infusion with midazolam 0.3 mg/kg/h.

Fetal death occurred* in utero* two days after admission, followed by expulsion of the fetus without hemorrhage. The renal function improved and urine output was 1 mL/kg/h after using furosemide 80 mg per day.

Ten days later, the patient was extubated after improvement of her neurological status. The laboratory test was then normalized. However, she kept sequelae: cerebellar ataxia and a severe peripheral polyneuropathy. The clinical examination showed slight decreased distal sensations and an important distal muscle weakness in both upper and lower extremities. The motor strength rated on a 0 to 5 scale [4] was at 2/5. Although she received intensive motor rehabilitation, the recovery from its motor deficit was slow. She was discharged from hospital two months after intoxication, while continuing motor rehabilitation program.

## 3. Discussion

The* P. harmala* is a shrub, 0.3–0.8 m tall with short creeping roots, white flowers, and round seed capsules carrying more than 50 seeds [1]. It is increasingly used for psychoactive recreational purposes. The pharmacologically active compounds of* P. harmala* include a number of *β*-carboline and quinazoline alkaloids. Harmaline, harmine, harmalol, harmol, and tetrahydroharmine were identified and quantified as the main *β*-carboline alkaloids in* P. harmala* extracts. Seeds and roots contained the highest levels of alkaloids with low levels in stems and leaves and absence in flowers. Harmine and harmaline are accumulated in dry seeds at 4.3% and 5.6%. Seed and roots extracts are potent reversible and competitive inhibitors of human monoamine oxidase (MAO-A) [5], which is main factor in degradation and reuptake of monoamines like serotonin and norepinephrine [1].

Quinazoline alkaloids (e.g., vasicine and vasicinone), within* P. harmala,* have been attributed to the abortifacient effect of this plant [6, 7]. In our case, the ingestion of seeds of* P. harmala* was in order to provoke abortion, which was realized spontaneously two days later.

In Morocco,* P. harmala* intoxication represents 4.6% of all plant intoxication [8]. It is used illegally as an abortifacient, due to the prohibition of abortion in Morocco except in rare life-threatening cases. It is also used in traditional medicine as sedative and soporific for insomniac and restless nursling. Ingestion is the main route of administration and intoxication, but inhalation and fumigation are also a common practice [9]. The symptomatology is dominated by neurological, gastrointestinal, and cardiovascular signs, respectively, 34.4%, 31.9%, and 15.8%. The mortality rate is about 6% [10].

In the cases of toxicity of* P. harmala* already reported [11–16], the neurological presentation is always prominent. All authors have reported decreasing level of consciousness from confusion to unconsciousness. It can also produce paralysis, visual hallucinations, euphoria, diffuse tremors, and convulsion. In our case, the deterioration of the GCS score to 10 and the hypercapnia indicated the intubation and the artificial ventilation. Our patient had also cerebellar ataxia, which is already reported in other intoxicated cases and was reversible [11, 16]. The particularity of our case is the persistence of unusual neurological sequelae two months after intoxication, including cerebellar ataxia and severe peripheral polyneuropathy.

All of the reported cases showed also digestive problems (nausea, vomiting) [11–16]; bradycardia has also been reported [11]. Liver injury already noted [13] was observed in our case; it was moderate and reversible. Kidney lesion was also reported in the literature [13, 15]; in our case, we noted a severe renal failure that needed initially hemodialysis and was reversible later.

The diagnosis of the intoxication is based on the recognition of the plant and the identification of alkaloids by high performance liquid chromatography, or by more sensitive method: the gas chromatography/mass spectrometry [10].

The intoxication context and the specific clinical presentation support* P. harmala *intoxication; however, the limitation of this case report is the lack of laboratory confirmation, due to the absence of adequate laboratory facility to detect alkaloids.

Emergency department doctors should recognize and treat this intoxication. In the absence of a specific antidote, rapid and adequate therapeutic support should be established. The treatment of* P. harmala* poisoning is mainly symptomatic, based on gastrointestinal decontamination (gastric lavage, activated charcoal), associated with the correction of organ failure and symptomatic treatment of digestive, cardiac, and neurological disorders. Evolution is generally favorable, but it can be fatal at very high doses [10].

## 4. Conclusion

*P. harmala* is used traditionally as an abortifacient agent in Morocco, North Africa, and the Middle East; therefore, the physicians working in this region must be familiar with clinical and biological signs of its toxicity, in order to establish early gastrointestinal decontamination. The prognosis of this intoxication is variable; most cases can be managed successfully, but in high doses of intoxication, evolution can be fatal.
